# 

**Published:** 1840-11

**Authors:** 


### CONTENTS

OF NO. X.

### ORIGINAL COMMUNICATIONS.

### ESSAYS AND CASES.

Art. I.—Reports of Cases treated in the Louisville Marine Hospital. By George W. Bayless, M. D., Demonstrator of Anatomy in the Medical Institute of Louisville. - 325

Art. II.—Stramonium in Dysmenorrhœa. By Dr. T. J. Cogley, of Mount Vernon, Ohio. - - - - - - - 338

Art. III.—Facts and Remarks on the Sensibility to Caloric. By Thomas Townsend, M. D., of Wheeling, Va. - - 340

Art. IV.—Intermittent Fever, Hepatitis, Pneumonitis, Laryngitis and Intestinal Mucous Irritation. By Daniel Drake, M. D., Professor of Clinical Medicine and Pathological Anatomy in the Medical Institute of Louisville. - 340

### REVIEWS.

Art. V.—1. A Memoir of the Life and Character of Philip Syng Physick, M. D. By John Randolph, M. D., Lecturer on Surgery, Member of the American Philosophical

Society, etc., etc. Philadelphia: T. K. & P. G. Collins. 1839. 8vo. pp. 114.

2. Necrological Notice of Dr. Philip Syng Physick; delivered before the American Philosophical Society, May 4th, 1838. By W. E. Horner, M. D., Professor of Anatomy in the University of Pennsylvania, etc., etc. Philadelphia: Haswell, Barrington & Haswell. 1838. 8vo. pp. 32. - - - - - - - - - 353

Art. VI.—Elements of Pathological Anatomy; illustrated by numerous engravings. By Samuel D. Gross, M. D. Boston: 1839. - - - - - - - - - 367

Art. VII.—Transactions of the Medical Society of the State of New York.—Parts II and III, Vol. IV. - - - 382

SELECTIONS FROM AMERICAN AND FOREIGN JOURNALS.

Ph. Ricord on the Special Treatment of Phimosis and Paraphimosis. Translated from the French for the Western Journal of Medicine and Surgery, by Benjamin Dennis, M. D., Cincinnati. - 393

The Rain for Nine Years. - - - - - - - - 396

ORIGINAL INTELLIGENCE.

Summer and Autumnal Diseases for 1840. - - - - - 399

Scarlet Fever. - - - - - - - - - - 400

Cow-Pox. - - - - - - - - - - - 400

Licking County Medical and Philosophical Society. - - - 401

Green County Medical Society. - - - - - - - 402

A Pin retained in the Body Nineteen Years and then discharged - 402

American Philosophical Society. - - - - - - - 403

Medical Society of Tennessee. - - - - - - - 404

Louisville Medical Institute. - - - - - - - 404

